# Supplementary material for: BAF complex-mediated chromatin relaxation is required for establishment of X chromosome inactivation
Source: Nat Commun. 2022 Mar 29;13:1658. doi: 10.1038/s41467-022-29333-1 (PMC8964718; doi:10.1038/s41467-022-29333-1)

SUPPLEMENTARY INFORMATION

Xmas mESC: A female embryonic stem cell system that reveals the BAF complex as a key regulator of establishment of X chromosome inactivation

Authors/Affiliations

Andrew Keniry[1,2,*], Natasha Jansz[1,2], Linden J. Gearing[1,2], Iromi Wanigasuriya[1,2], Joseph Chen[3,4,5], Christian M. Nefzger[3,6], Peter F. Hickey[1,2], Quentin Gouil[1,2], Joy Liu[1,2], Kelsey A. Breslin[1], Megan Iminitoff[1,2], Tamara Beck[1], Andres Tapia del Fierro[1,2], Lachlan Whitehead[1,2], Andrew Jarratt[1,2], Sarah A. Kinkel[1,2], Phillippa C. Taberlay[7], Tracy Willson[1,2], Miha Pakusch[1], Matthew E. Ritchie[1,2], Douglas J. Hilton[1,2], Jose M. Polo[3,4,5] and Marnie E. Blewitt[1,2,*]

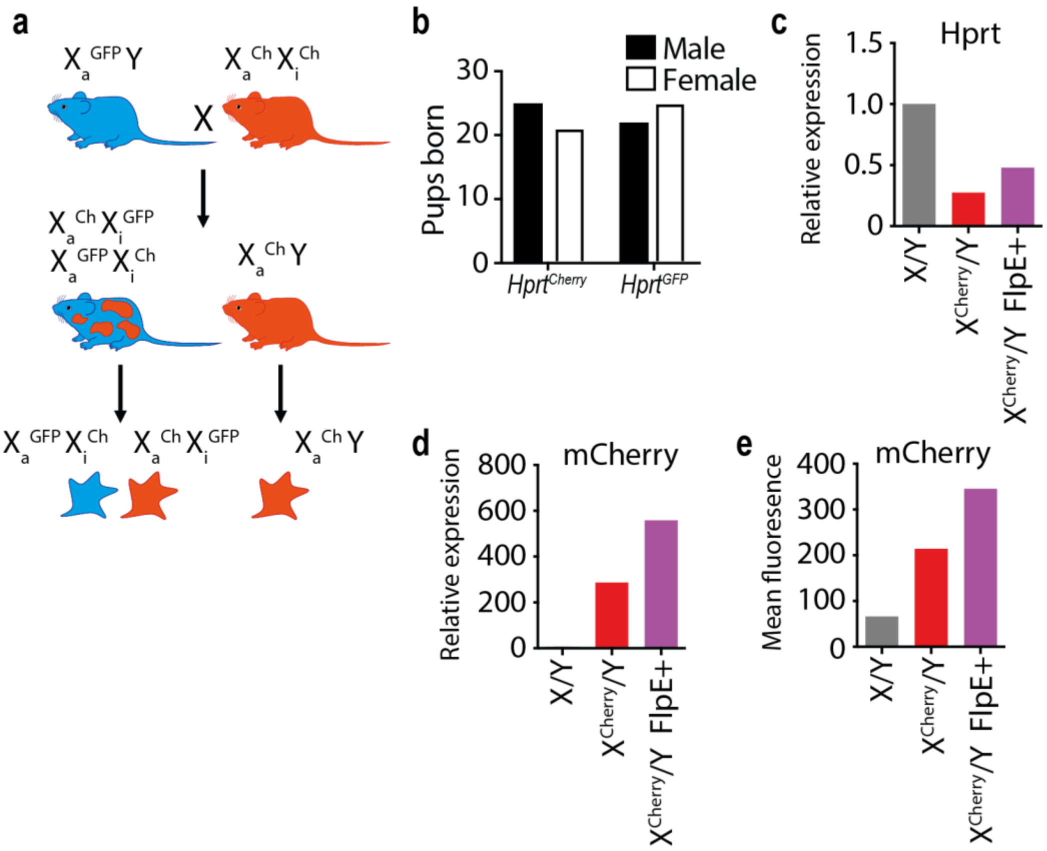

Supplementary Figure 1. Xmas reporter alleles are well tolerated

(a) Colour blind friendly version of Fig. 1b. mCherry is presented in orange and GFP in blue. **(b)** Numbers of male and female mice born of the indicated homozygous/hemizygous genotypes. **(c)** *Hprt* expression measured by qRT-PCR in mESCs of the indicated genotypes. **(d)** mCherry expression measured by qRT-PCR in mESCs of the indicated genotypes. **(e)** mCherry expression measured by flow cytometry in mESCs of the indicated genotypes. Source data are provided as a source data file.

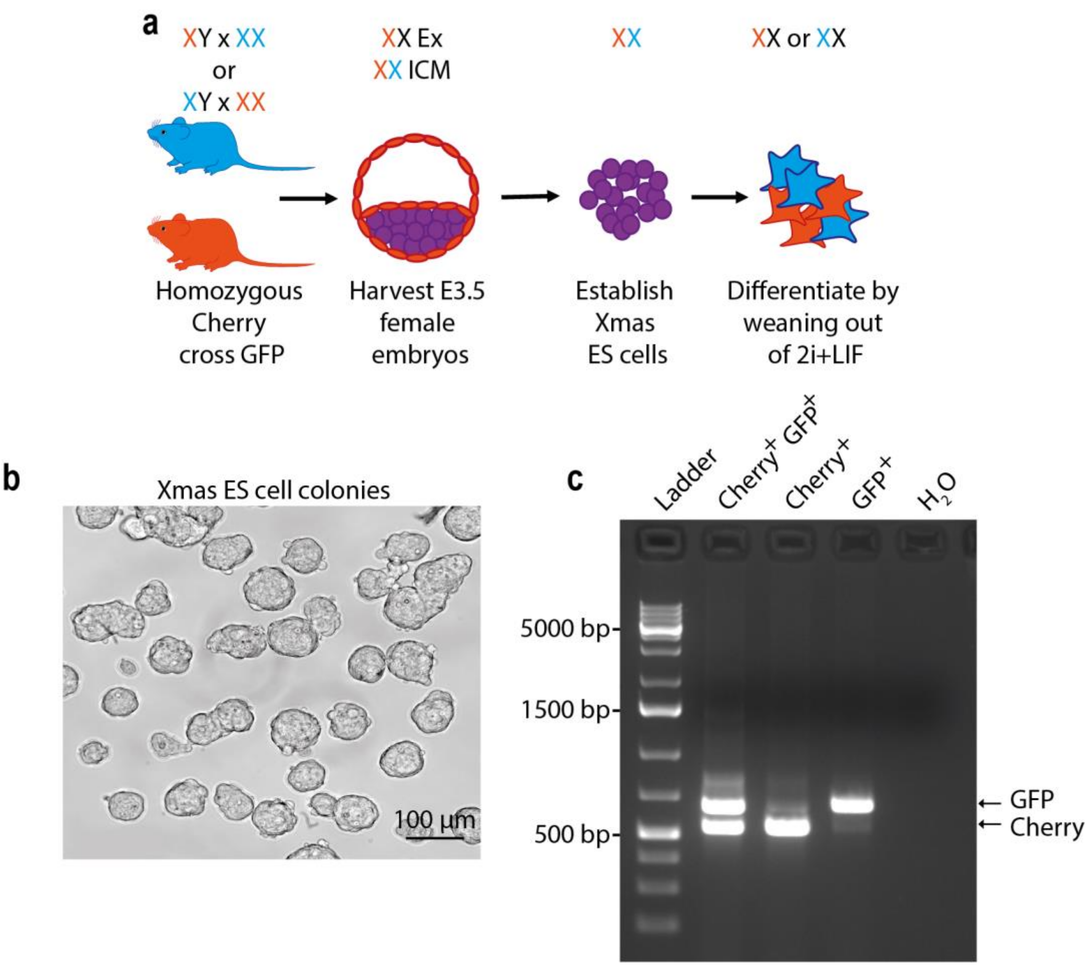

Supplementary Figure 2. Xmas mESCs allow development of improved culture conditions.

(a) Colour blind friendly version of Fig.2a. mCherry is presented in orange, GFP in blue and mixed mCherry and GFP in purple. **(b)** Bright field microscopy image of Xmas mESC colonies maintained under our improved culture conditions. **(c)** Gel electrophoresis of PCR product of the fluorescent reporter constructs produced from DNA of Xmas mESCs purified by FACS into GFP^{+}, mCherry^{+} and Cherry^{+}GFP^{+} double positive populations. Images shown are from a single experiment.

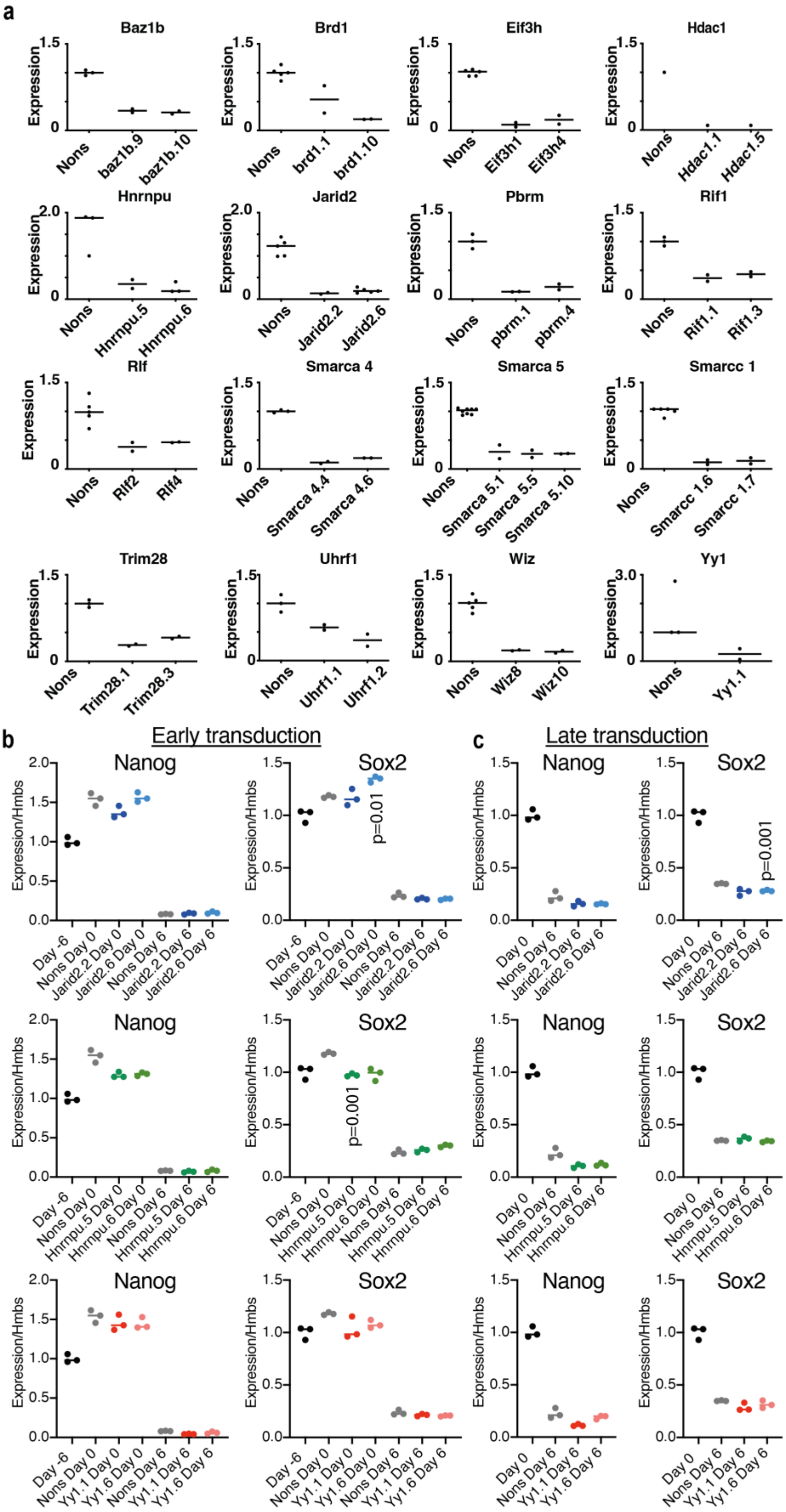
a
Baz1b
Brd1
Eif3h
Hdac1
Expression
1.5
0
Nons
baz1b.9
baz1b.10
brd1.1
brd1.10
Eif3h1
Eif3h4
Hdac1.1
Hdac1.5
Hnrnpu
Jarid2
Pbrm
Rif1
2.0
Hnrnpu.5
Hnrnpu.6
Jarid2.2
Jarid2.6
pbrm.1
pbrm.4
Rif1.1
Rif1.3
Rlf
Smarca 4
Smarca 5
Smarcc 1
Rlf2
Rlf4
Smarca 4.4
Smarca 4.6
Smarca 5.1
Smarca 5.5
Smarca 5.10
Smarcc 1.6
Smarcc 1.7
Trim28
Uhrf1
Wiz
Yy1
3.0
Trim28.1
Trim28.3
Uhrf1.1
Uhrf1.2
Wiz8
Wiz10
Yy1.1
b
Early transduction
c
Late transduction
Nanog
Sox2
Expression/Hmbs
p=0.01
p=0.001
Day -6
Nons Day 0
Jarid2.2 Day 0
Jarid2.6 Day 0
Nons Day 6
Jarid2.2 Day 6
Jarid2.6 Day 6
Day 0
Hnrnpu.5 Day 0
Hnrnpu.6 Day 0
Hnrnpu.5 Day 6
Hnrnpu.6 Day 6
Yy1.1 Day 0
Yy1.6 Day 0
Yy1.1 Day 6
Yy1.6 Day 6

Supplementary Figure 3. Hairpin and Xmas differentiation validation.

(a) Bar graphs showing the expression of the indicated genes relative to *Hmbs*, measured by qRT-PCR following knockdown with the indicated shRNAs. Knockdown was measured in either mESCs or MEFs three days following viral transduction of shRNA (n = 1 to 3 independent replicates, error bars indicate s.e.m.). **(b)** qRT-PCR of Nanog and Sox2 at day 0 and day 6 of differentiation in Xmas mESCs when transduced with the indicated shRNA 6 days prior to induction of differentiation (Early transduction). n = 3 independent replicates. Two-tailed unpaired Student's *t*-test with multiple testing correction. Only statistically significant *p*-values are given. **(c)** qRT-PCR of Nanog and Sox2 at day 0 and day 6 of differentiation in Xmas mESCs when transduced with the indicated shRNA 2 days post induction of differentiation (Late transduction). n =3 independent replicates. Two-tailed unpaired Student's *t*-test with multiple testing correction. No statistically significant *p*-values were obtained. Source data are provided as a source data file.

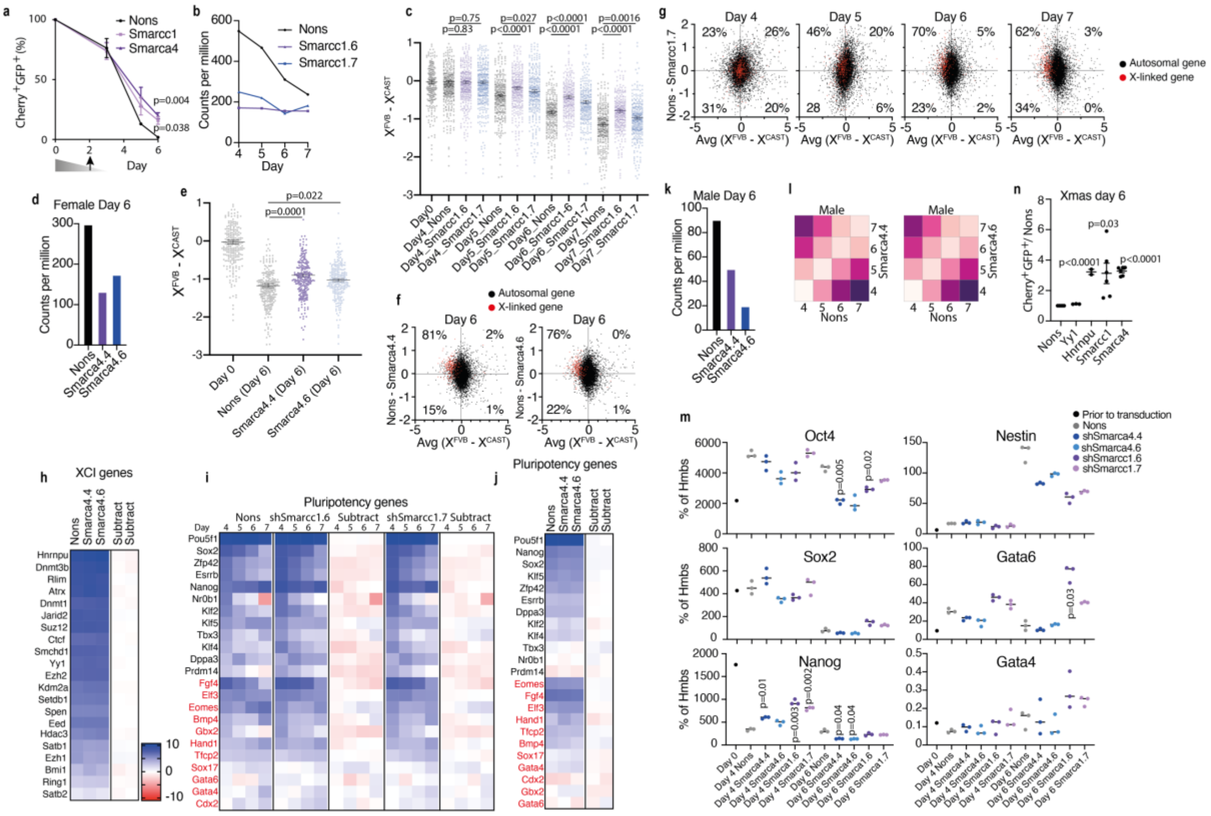

Supplementary Figure 4. *Smarcc1* and *Smarca4* depletion cause failure of XCI.

(a) Flow cytometry data along a time course of Xmas mESC differentiation following shRNA mediated knockdown of *Smarcc1*, *Smarca4* or Nons (n = 4 independent replicates with two shRNAs per gene, error bars indicate s.e.m., Two-way ANOVA, *p*-values are given. **(b)** RNA-seq of differentiating $X^{FVB}X^{CAST}$ mESCs, showing expression (log_2 rpm) of *Smarcc1* following its knockdown. **(c)** Expanded version of data from Fig. 5d showing allele specific RNA-seq of differentiating $X^{FVB}X^{CAST}$ mESCs following knockdown of *Smarcc1*. Two-tailed Student's unpaired *t*-test, n = 246 to 371 informative genes, *p*-values are given. **(d)** RNA-seq of differentiating $X^{FVB}X^{CAST}$ mESCs, showing expression (rpm log_2) of *Smarca4* following its knockdown. **(e)** Expanded version of data from Fig. 5e showing allele specific RNA-seq of differentiating $X^{FVB}X^{CAST}$ mESCs following knockdown of *Smarca4*. Error bars show mean+/-SEM. Two-tailed Student's unpaired *t*-test, n = 239 to 254 informative genes, *p*-values are given. **(f,g)** These graphs show RNA-seq data and are designed to compare gene expression from the X chromosome with respect to autosomes. Each point on the graph represents an informative gene, with X-linked genes in red and autosomal genes in black. The x-axis shows the ratio of expression from FVB compared to CAST (X^{FVB}-X^{CAST} log_2), therefore XCI is

observed as a left shift of the red dots along the x-axis. The y-axis shows the ratio of expression from Nons compared to knockdown with *Smarca4* **(f)** or *Smarcc1* **(g)** (log_2FC rpm Nons − knockdown), therefore a failure of XCI in the knockdown is observed as an upward shift of the red dots along the y-axis. Black dots give an indication of global trends in gene expression. Dotted lines indicate medians and percentages show the X-linked genes falling into each quadrant. **(h,i,j)** Heat maps of gene expression (rpm log_2) with the difference between knockdown and control (subtract, Nons – knockdown) indicated for known XCI regulators following Smarca4 knockdown **(h)** and pluripotency and differentiation genes following Smarcc1 **(i)** or Smarca4 **(j)** knockdown. Pluripotency associated genes appear in black type, differentiation associated genes in red. Scale bar represents both log_2 rpm and log_2FC (h-j). **(k)** RNA-seq of differentiating male mESCs, showing expression (rpm log_2) of *Smarca4* following its knockdown. **(l)** Heat maps showing the average Euclidean distance in gene expression (log_2 rpm) between *Smarca4* knockdown and Nons control along a differentiation time course in male mESCs. **(m)** qRT-PCR in female mESCs at day 4 and day 6 of differentiation following knockdown with the indicated hairpins for pluripotency genes (Oct4, Sox2, Nanog) or differentiation genes (Nestin, Gata4, Gata6). Two-tailed Student's unpaired *t*-test with multiple testing correction, only significant *p*-values are given, n = 3 independent replicates. **(n)** Xmas mESCs transduced with the indicated hairpins on day 3 of differentiation, with fluorescence measured by flow cytometry at day 6 (n = 2 to 6 independent replicates, error bars show the s.e.m, Two-tailed Student's unpaired *t*-test, *p*-values are given. Source data are provided as a source data file.

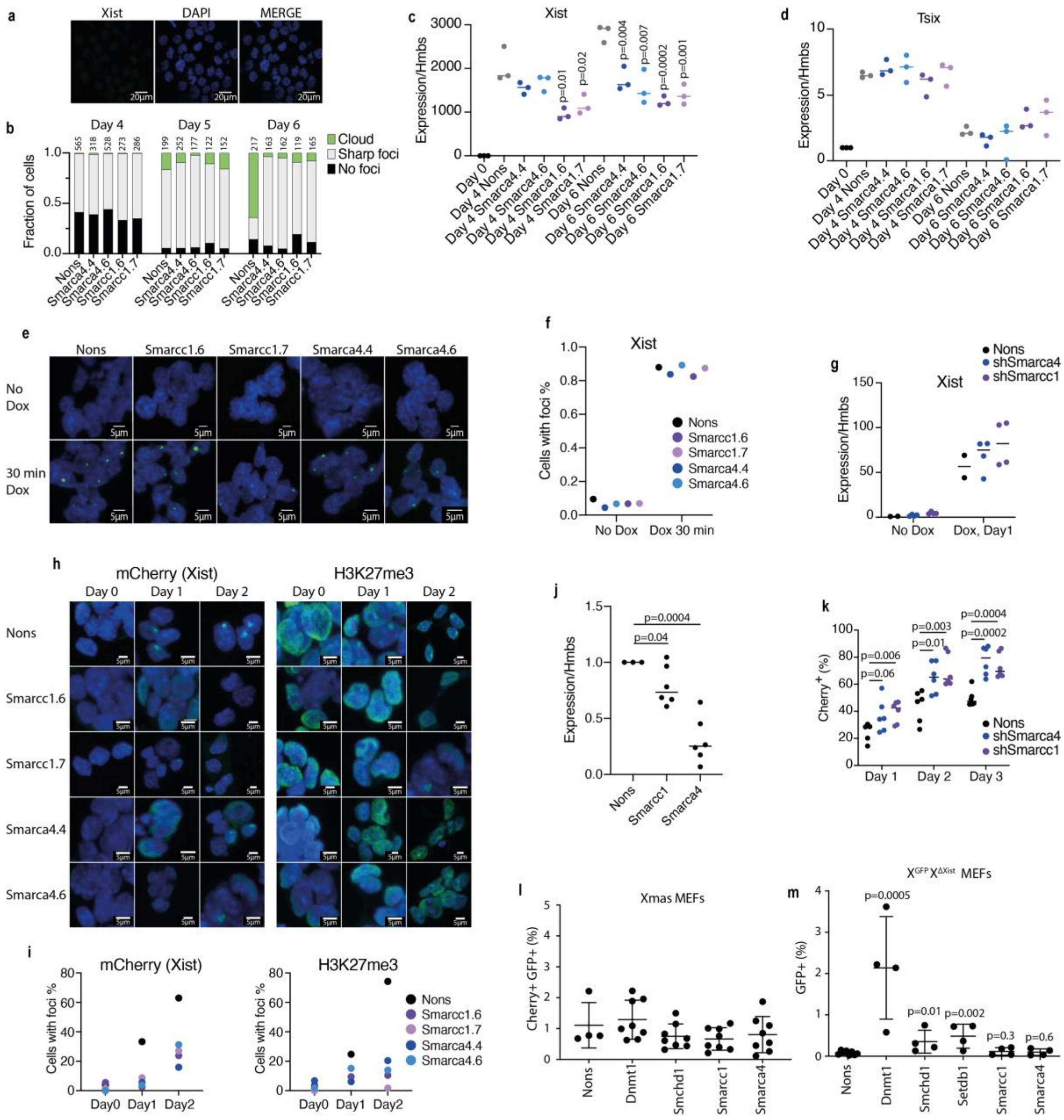

Supplementary Figure 5. Smarcc1 and Smarca4 are required to establish the Xi.

(a) RNA FISH for *Xist* in female mESCs at day 0 of differentiation. *Xist* staining in green, DAPI in blue. Representative images from a single experiment are shown. **(b)** Quantification of data from (6a). **(c,d)** qRT-PCR in female mESCs at day 4 and day 6 of differentiation following knockdown with the indicated hairpins for *Xist* **(c)** and *Tsix* **(d)**. Two-tailed Student's unpaired *t*-test, only significant *p*-values are given, n = 3 independent replicates. **(e-k)** Show experiments in male mESCs with inducible *Xist*:BglSL-mCherry[75]. *Xist* expression is induced from chromosome 17 by the addition of doxycycline and can be detected by an antibody against mCherry. Cells were challenged with either *Smarcc1*, *Smarca4* or nonsilencing control

knockdown, as indicated. Representative images from each experiment are shown. **(e)** RNA FISH for *Xist* prior to (No Dox) or following 30 minutes of *Xist* induction with doxycycline. *Xist* staining in green, DAPI in blue. **(f)** Quantification of data from (6e). **(g)** qRT-PCR for *Xist* before or one day post doxycycline induction. Two-tailed Student's unpaired *t*-test, n = 2 to 4 independent replicates, no statistically significant results were obtained. **(h)** Immunostaining for mCherry (*Xist)* or H3K27me3 at day 0, 1 or 2 post *Xist* induction, shown in green. DAPI staining in blue. **(i)** Quantification of data shown in (6h). **(j)** qRT-PCR for *Xist* following 24 hours of doxycycline induction then 24 hours of washout, allowing measurement of *Xist* transcript stability. One-tailed Student's unpaired *t*-test, *p*-values are given, n = 3 to 6 independent replicates with two hairpins per gene. **(k)** Flow cytometry data for mCherry at days 1, 2 and 3 post *Xist* induction. Note that cells that undergo *Xist* induced gene silencing will die, therefore mCherry positivity is a measure of failed gene silencing. Two-tailed Student's unpaired *t*-test, *p*-values are given, n = 6 independent replicates with two hairpins per gene. **(l)** Flow cytometry data from Xmas MEFs following knockdown of indicated gene and treatment with 5-azacytidine. **(m)** Flow cytometry data from $Xi^{GFP}Xa$ MEFs following knockdown of indicated gene and treatment with 5-azacytadine. (l, m) n = 4 to 8 independent replicates with two hairpins per gene and error bars show the s.e.m., Two-tailed Student's unpaired *t*-test, *p*-values are given. Source data are provided as a source data file.

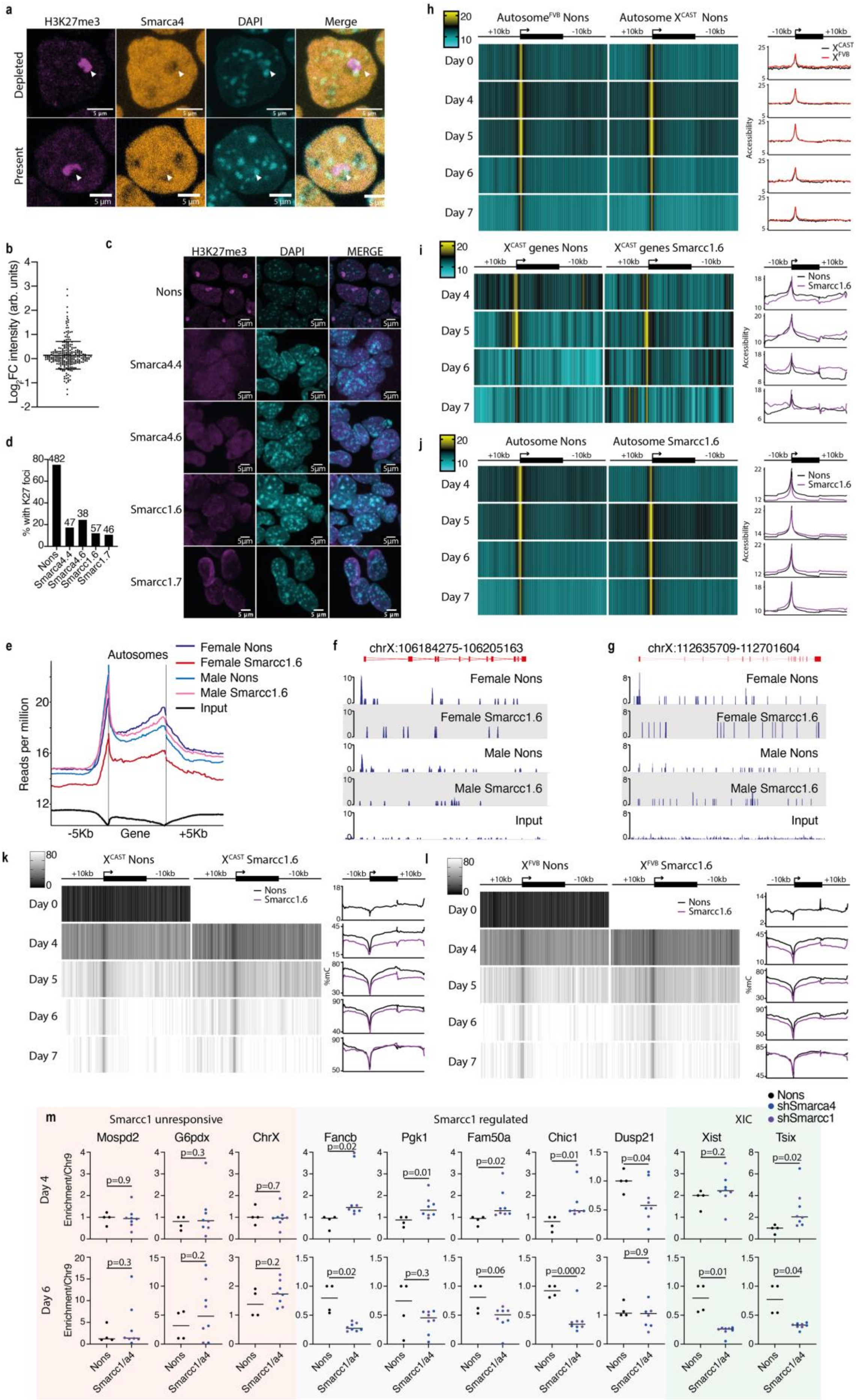
a
H3K27me3
Smarca4
DAPI
Merge
Depleted
Present
5 µm
b
Log2FC intensity (arb. units)
c
H3K27me3
DAPI
MERGE
Nons
Smarca4.4
Smarca4.6
Smarcc1.6
Smarcc1.7
d
% with K27 foci
482
47
38
57
46
Nons
Smarca4.4
Smarca4.6
Smarcc1.6
Smarcc1.7
e
Autosomes
Female Nons
Female Smarcc1.6
Male Nons
Male Smarcc1.6
Input
Reads per million
-5Kb
Gene
+5Kb
f
chrX:106184275-106205163
Female Nons
Female Smarcc1.6
Male Nons
Male Smarcc1.6
Input
g
chrX:112635709-112701604
Female Nons
Female Smarcc1.6
Male Nons
Male Smarcc1.6
Input
h
Autosome FVB Nons
Autosome X CAST Nons
+10kb
-10kb
Day 0
Day 4
Day 5
Day 6
Day 7
X CAST
X FVB
Accessibility
i
X CAST genes Nons
X CAST genes Smarcc1.6
Nons
Smarcc1.6
Day 4
Day 5
Day 6
Day 7
Accessibility
j
Autosome Nons
Autosome Smarcc1.6
Nons
Smarcc1.6
Day 4
Day 5
Day 6
Day 7
Accessibility
k
X CAST Nons
X CAST Smarcc1.6
Nons
Smarcc1.6
Day 0
Day 4
Day 5
Day 6
Day 7
%mC
l
X FVB Nons
X FVB Smarcc1.6
Nons
Smarcc1.6
Day 0
Day 4
Day 5
Day 6
Day 7
%mC
m
Nons
shSmarca4
shSmarcc1
Smarcc1 unresponsive
Smarcc1 regulated
XIC
Mospd2
G6pdx
ChrX
Fancb
Pgk1
Fam50a
Chic1
Dusp21
Xist
Tsix
Day 4
Day 6
Enrichment/Chr9
p=0.9
p=0.3
p=0.7
p=0.02
p=0.01
p=0.02
p=0.01
p=0.04
p=0.2
p=0.02
p=0.3
p=0.2
p=0.2
p=0.02
p=0.3
p=0.06
p=0.0002
p=0.9
p=0.01
p=0.04
Nons
Smarcc1/a4

Supplementary Figure 6. Smarcc1 and Smarca4 are not required for maintenance of XCI.

(a) Immunostaining of H3K27me3 and Smarca4 in female mESCs at day 6 of differentiation. Representative images of Smarca4 being both present and depleted at sites of H3K27me3 are shown. White arrows indicate H3K27me3 foci. **(b)** Plot of data shown in (a) indicating the average intensity of Smarca4 in arbitrary units (arb. units) underneath H3K27me3 foci compared to the rest of the nucleus (log_2FC), for all cells measured. Error bars represent the mean +/- standard deviation. **(c)** Immunostaining of H3K27me3 in female mESCs at day 6 of differentiation following knockdown with the indicated hairpins. **(d)** Quantification of data shown in (c). **(e-g)** Smarca4 ChIP-seq in male and female mESCs at day 4 of differentiation, with either a Smarcc1 knockdown or nonsilencing control. **(e)** shows the average read densty across all autosomal genes including 5kb up and downstream and **(f,g)** show example coverage plots. **(h,i,j)** Nucleosome occupancy (% GpC methylation) along a time course of female mESC differentiation determined by NOMe-seq averaged across all genes and flanking regions on autosome FVB and CAST genes **(h),** or the X^{CAST} **(i)** or autosomes **(j)** upon Smarcc1.6 knockdown. The data shown as a heat map or a smoothed histogram. **(k,l)** DNA methylation (% CpG methylation) along a time course of female mESC differentiation determined by NOMe-seq averaged across all genes and flanking regions on the X^{CAST} **(k)** or X^{FVB} **(l)** upon Smarcc1.6 knockdown. The data shown as a heat map or a smoothed histogram. **(m)** MARs-qPCR data shown for promoters of genes that were either unresponsive or responsive to Smarcc1 knockdown from the NOMe-seq data, in female Xmas mESCs at either day 4 or day 6 of differentiation, with either *Smarcc1*, *Smarca4* or nonsilencing control knockdown. The promoters of *Xist* and *Tsix* are also shown. Note that a higher enrichment value indicates higher nucleosome density. *Smarca4* and *Smarcc1* depleted samples were pooled for statistical analysis, Two-tailed Welch's unpaired *t*-test, *p*-values are given. n = 4 independent replicates per gene with two hairpins per gene. Source data are provided as a source data file.

Xmas mESC Gating Strategy

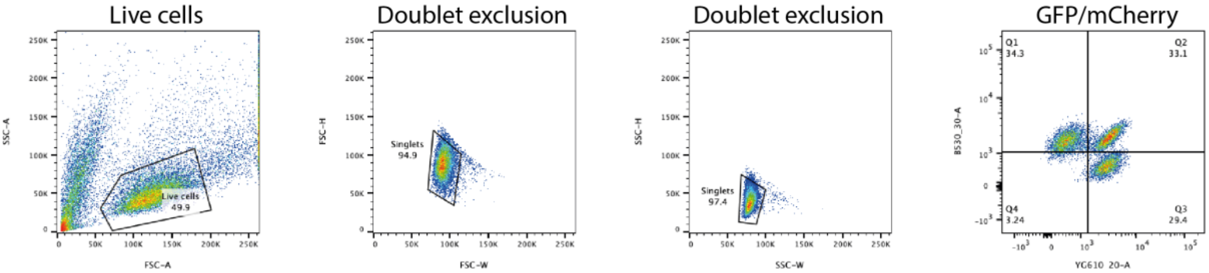

Supplement: Supplementary file 1 — Supplementary information [file 41467_2022_29333_MOESM1_ESM.pdf]
