# Supplementary material for: BAF complex-mediated chromatin relaxation is required for establishment of X chromosome inactivation
Source: Nat Commun. 2022 Mar 29;13:1658. doi: 10.1038/s41467-022-29333-1 (PMC8964718; doi:10.1038/s41467-022-29333-1)
Supplement: Supplementary file 3 — Description of additional Supplementary File [file 41467_2022_29333_MOESM3_ESM.pdf]

Descriptions of Additional Supplementary Information Files

Supplementary Data 1: RNA-seq in differentiating Xmas mESCs Table provides analysed RNA-seq data along a timecourse of Xmas mESC differentiation. Expression values are given in rpm log2.

Supplementary Data 2: RNA-seq in female mESC with Smarca4 knockdown Table provides allele specific RNA-seq data at day6 of FVB cross CAST female mESC differentiation with either Smarca4 knockdown or Nons control. Values given are the XFVBXCAST log2 value for only informative genes.

Supplementary Data 3: Allele split RNA-seq in female mESC with Smarcc1 knockdown Table provides allele specific RNA-seq data along a timecourse of FVB cross CAST female mESC differentiation with either Smarcc1 knockdown or Nons control. Values given are the XFVB-XCAST log2 value for only informative genes.

Supplementary Data 4: RNA-seq in female mESC with Smarcc1 knockdown Table RNA-seq data along a timecourse of FVB cross CAST female mESC differentiation with either Smarcc1 knockdown, Smarca4 knockdown or Nons control. Values given are log2CPM.

Supplementary Data 5: RNA-seq in male mESC Table provides analysed RNA-seq data along a timecourse of male mESC differentiation. Expression values are given in rpm log2.

Supplementary Data 6 : Oligonucleotides - Table provides the sequences of oligonucleotides used in this study for qRT-PCR, genotyping and shRNA knockdown.
