# Supplementary material for: BAF complex-mediated chromatin relaxation is required for establishment of X chromosome inactivation
Source: Nat Commun. 2022 Mar 29;13:1658. doi: 10.1038/s41467-022-29333-1 (PMC8964718; doi:10.1038/s41467-022-29333-1)
Supplement: Supplementary file 10 — Reporting Summary [file 41467_2022_29333_MOESM10_ESM.pdf]

nature research

Corresponding author(s): Andrew Keniry
Marnie Blewitt

Last updated by author(s): Feb 2, 2022

Reporting Summary

Nature Research wishes to improve the reproducibility of the work that we publish. This form provides structure for consistency and transparency in reporting. For further information on Nature Research policies, see Authors & Referees and the Editorial Policy Checklist.

Statistics

For all statistical analyses, confirm that the following items are present in the figure legend, table legend, main text, or Methods section.

n/a	Confirmed	
☐	☒	The exact sample size (*n*) for each experimental group/condition, given as a discrete number and unit of measurement
☐	☒	A statement on whether measurements were taken from distinct samples or whether the same sample was measured repeatedly
☐	☒	The statistical test(s) used AND whether they are one- or two-sided<br>*Only common tests should be described solely by name; describe more complex techniques in the Methods section.*
☒	☐	A description of all covariates tested
☐	☒	A description of any assumptions or corrections, such as tests of normality and adjustment for multiple comparisons
☐	☒	A full description of the statistical parameters including central tendency (e.g. means) or other basic estimates (e.g. regression coefficient) AND variation (e.g. standard deviation) or associated estimates of uncertainty (e.g. confidence intervals)
☐	☒	For null hypothesis testing, the test statistic (e.g. *F*, *t*, *r*) with confidence intervals, effect sizes, degrees of freedom and *P* value noted<br>*Give P values as exact values whenever suitable.*
☒	☐	For Bayesian analysis, information on the choice of priors and Markov chain Monte Carlo settings
☒	☐	For hierarchical and complex designs, identification of the appropriate level for tests and full reporting of outcomes
☒	☐	Estimates of effect sizes (e.g. Cohen's *d*, Pearson's *r*), indicating how they were calculated

Our web collection on statistics for biologists contains articles on many of the points above.

Software and code

Policy information about availability of computer code

Data collection	No software was used.
Data analysis	Seqmonk v1.36.1 and v1.38.2 was used to visualise and perform statistical analysis on all RNAseq, ChIP-seq and NOMe-seq datasets.<br>SNPsplit_ v0.3.2 was used to process allele specific RNA-seq and NOMe-Seq<br>bowtie2 v2.2.5 was used to map RNA-seq<br>Hisat2 v2.0.5 was used to map RNA-seq<br>tophat v2.1.1 was used to map ChIP-seq<br>trim_galore v0.4.4 was used for adapter trimmed<br>voom v3.40.6 was used to analyse RNA-seq<br>SNPsplit v0.3.2 was used to haplotype sequencing data<br>edgeR v3.26.7 was used to analyse RNA-seq<br>EGSEA v1.12.0 was used for gene set testing<br>R v3.6.1 was used for Euclidean distance calculations<br>Fiji (ImageJ2 v 2.3.0/1.53n) was used to analyse microscopy data.<br>Custom Fiji macro for analysing colocolisation between two channels. Available here https://github.com/DrLachie/smchd1_coloc<br>FloJo v9.9.6 was used for analysis of flow cytometry data

For manuscripts utilizing custom algorithms or software that are central to the research but not yet described in published literature, software must be made available to editors/reviewers. We strongly encourage code deposition in a community repository (e.g. GitHub). See the Nature Research guidelines for submitting code & software for further information.

Data

Policy information about availability of data

All manuscripts must include a data availability statement. This statement should provide the following information, where applicable:

- Accession codes, unique identifiers, or web links for publicly available datasets
- A list of figures that have associated raw data
- A description of any restrictions on data availability

All next generation sequencing data generated for this project have been deposited in the Gene Expression Omnibus (GEO) database under accession number GSE137163 (https://www.ncbi.nlm.nih.gov/geo/query/acc.cgi?acc=GSE137163). Publicly available data was utilised in this study and is available from the GEO database under accession numbers GSE23943 (https://www.ncbi.nlm.nih.gov/geo/query/acc.cgi?acc=GSE23943) and GSE67299 (https://www.ncbi.nlm.nih.gov/geo/query/acc.cgi?acc=GSE67299). Source data are provided with this paper.

Field-specific reporting

Please select the one below that is the best fit for your research. If you are not sure, read the appropriate sections before making your selection.

☒ Life sciences ☐ Behavioural & social sciences ☐ Ecological, evolutionary & environmental sciences

For a reference copy of the document with all sections, see nature.com/documents/nr-reporting-summary-flat.pdf

Life sciences study design

All studies must disclose on these points even when the disclosure is negative.

Sample size	The majority of experiments were performed on n>3, to ensure reproducibility while being affordable. Some genomic data is provided as less than n = 3 to reduce costs, however statistical power is obtained by analysing the X choromsome as a whole or through multiple time points in a time course. Sample sizes necessary for a particular experiment were chosen based on prior knowledge.
Data exclusions	No data was excluded
Replication	The majority of experiments were replicated. Additionally, in multiple techniques were performed to address questions. Replicates where positive controls failed were excluded.
Randomization	It was not appropriate to randomise samples. Covariates were controlled by comparison to the most appropriate negative controls.
Blinding	The scoring of microscope images and flow cytometry data was blinded. For other successful completion of other experiments it was necessary for the operator to be unblinded.

Reporting for specific materials, systems and methods

We require information from authors about some types of materials, experimental systems and methods used in many studies. Here, indicate whether each material, system or method listed is relevant to your study. If you are not sure if a list item applies to your research, read the appropriate section before selecting a response.

Materials & experimental systems

n/a	Involved in the study	
☐	☒	Antibodies
☐	☒	Eukaryotic cell lines
☒	☐	Palaeontology
☐	☒	Animals and other organisms
☒	☐	Human research participants
☒	☐	Clinical data

Methods

n/a	Involved in the study	
☐	☒	ChIP-seq
☐	☒	Flow cytometry
☒	☐	MRI-based neuroimaging

Antibodies

Antibodies used

BUV395 Thy1.2 BD Biosciences 565257, 1:200 dilution.
BV421 EpCAM BD Biosciences 563214, 1:100 dilution.
SSEA1-Biotin eBioscience 13-8813-82, 1:400 dilution.
Streptavidin Pe-Cy7 BD Biosciences 557598, 1:200 dilution.
Smarca4 Abcam ab110641, 1:100 dilution.
H3K27me3 Millipore 07-449, 1:100 dilution.
H3K27me3 Cell Signalling Technology C36B11, 1:100 dilution.
Smarcc1 anti-rabbit IgG Cell Signaling #11956S, 1:100 dilution.

Smarca4 (for ChIP) Cell Signalling Technology D1Q7F, 10ug per IP.
mCherry Novus Biologicals NBP2-25158, 1:100 dilution.
PE-Cy7-conjugated anti-Sca1 BD Pharmingen Clone D7 (RUO), 1:100 dilution.
Ter119, B220, CD19, Gr1, CD2, CD3 and CD8, APC-conjugated anti-c-kit/CD117 were generated in-house by the WEHI antibody facility. These antibodies used at 1:100 dilution.

Validation

We validated that each antibody behaved as expected in our controls for each experiment. Antibodies used for IF were confirmed to produce the expected staining patterns, either with nuclear localisation or the presence of a foci at the inactive X chromosome. Antibodies used for flow cytometry were confirmed to identify the expected cell population. All commercial antibodies have validation data available from the supplier websites, as follows.

BUV395 Thy1.2 BD Biosciences 565257
https://www.bdbiosciences.com/en-au/products/reagents/flow-cytometry-reagents/research-reagents/single-color-antibodies-ruo/buv395-rat-anti-mouse-cd90-2.565257

BV421 EpCAM BD Biosciences 563214
https://www.bdbiosciences.com/en-au/products/reagents/flow-cytometry-reagents/research-reagents/single-color-antibodies-ruo/bv421-mouse-anti-human-cd326.563180

SSEA1-Biotin eBioscience 13-8813-82
file:///Users/keniry/Downloads/Antibody-SSEA1.pdf

Streptavidin Pe-Cy7 BD Biosciences 557598
https://www.bdbiosciences.com/en-au/products/reagents/flow-cytometry-reagents/research-reagents/single-color-antibodies-ruo/streptavidin-apc-cy-7.554063

Smarca4 Abcam ab110641
https://www.abcam.com/brg1-antibody-epncir111a-ab110641.html

H3K27me3 Millipore 07-449
https://www.merckmillipore.com/AU/en/product/Anti-trimethyl-Histone-H3-Lys27-Antibody,MM_NF-07-449

H3K27me3 Cell Signalling Technology C36B11
https://www.cellsignal.com/products/antibody-conjugates/tri-methyl-histone-h3-lys27-c36b11-rabbit-mab-alexa-fluor-647-conjugate/12158

Smarcc1 anti-rabbit IgG Cell Signaling #11956S
https://www.cellsignal.com/products/primary-antibodies/smarcc1-baf155-d7f8s-rabbit-mab/11956

Smarca4 (for ChIP) Cell Signalling Technology D1Q7F
https://www.cellsignal.com/products/primary-antibodies/brg1-d1q7f-rabbit-mab/49360

mCherry Novus Biologicals NBP2-25158
https://www.novusbio.com/products/mcherry-antibody_nbp2-25158

PE-Cy7-conjugated anti-Sca1 BD Pharmingen Clone D7
https://www.bdbiosciences.com/en-au/products/reagents/flow-cytometry-reagents/research-reagents/single-color-antibodies-ruo/pe-cy-7-rat-anti-mouse-ly-6a-e.561021

Eukaryotic cell lines

Policy information about cell lines

Cell line source(s)

Xmas ESCs derived from XHprt-GFP and XHprt-mCherry crosses
Xmas MEFs derived from XHprt-GFP and XHprt-mCherry crosses
F1 ESCs derived from FVB and CAST crosses
MEFs derived from D4/XEGFP and XistΔA crosses
Male mESCs with inducible Xist:BglII:Cherry on chromosome 17 (Moindrot et al., Cell Rep, 2015)

Authentication

These cell lines were authenticated based on control samples behaving as expected.

Mycoplasma contamination

All cell lines were derived fresh for this study, often multiple times, and therefore were not at risk of mycoplasma. They were therefore not tested for such.

Commonly misidentified lines
(See ICLAC register)

No commonly misidentified lines were used.

Animals and other organisms

Policy information about studies involving animals; ARRIVE guidelines recommended for reporting animal research

Laboratory animals

XHprt-GFP XHprt-GFP mouse strain (Xmas C57/Bl6), males and females used for breeding and retired at approximately 1 year of age.

	XHprt-mCherry XHprt-mCherry mouse strain (Xmas C57/Bl6), males and females used for breeding and retired at approximately 1 year of age.<br>Castaneus (CAST/EiJ), males and females used for breeding and retired at approximately 1 year of age.<br>FVB/NJ, males and females used for breeding and retired at approximately 1 year of age.<br>D4/XEGFP mouse strain (Hadjantonakis et al., 1998), males and females used for breeding and retired at approximately 1 year of age.<br>XistΔA mouse strain (Royce-Tolland et al., 2010), males and females used for breeding and retired at approximately 1 year of age.
Wild animals	No wild animals used in this study.
Field-collected samples	No field-collected samples used in this study.
Ethics oversight	Animals were housed and treated according to Walter and Eliza Hall Institute (WEHI) Animal Ethics Committee approved protocols (2014.034, 2018.004).

Note that full information on the approval of the study protocol must also be provided in the manuscript.

ChIP-seq

Data deposition

☒ Confirm that both raw and final processed data have been deposited in a public database such as GEO.

☒ Confirm that you have deposited or provided access to graph files (e.g. BED files) for the called peaks.

Data access links<br>*May remain private before publication.*	Gene Expression Omnibus, under GSE137163. https://www.ncbi.nlm.nih.gov/geo/query/acc.cgi?acc=GSE137163
Files in database submission	Brg1-Female-Nons_S1_R1.merge.fastq.gz<br>Brg1-Female-Smarcc1-6_S2_R1.merge.fastq.gz<br>Brg1-Male-Nons_S3_R1.merge.fastq.gz<br>Brg1-Male-Smarcc1-6_S4_R1.merge.fastq.gz<br>Input-Nons_S15_R1.fastq.gz<br>Macs2_peaks.xlsx
Genome browser session (e.g. UCSC)	No longer applicable.

Methodology

Replicates	Single replicate
Sequencing depth	Brg1-Female-Nons: 87952953 reads , 3031128 uniquely mapped reads<br>Brg1-Female-Smarcc1-6: 80969125 reads , 1560849 uniquely mapped reads<br>Brg1-Male-Nons: reads 280343197 , 5686343 uniquely mapped reads<br>Brg1-Male-Smarcc1-6: 30478300 reads , 3711184 uniquely mapped reads<br>Input: reads 79916967, 26073233 uniquely mapped reads
Antibodies	10ug Smarca4 (for ChIP) Cell Signalling Technology D1Q7F
Peak calling parameters	tophat was used to map reads to mm10. The macs2 peak caller housed within the Seqmonk software was used to call peaks at an FDR below 5% for a fragment size of 300bp. The Input sample was used as the control.
Data quality	ChIP-seq antibody was validated by the appearance of identifiable and statistically significant peaks.<br>Brg1-Female-Nons: 21245 peaks called below 5% FDR and above 5-fold enrichment.<br>Brg1-Female-Smarcc1-6: 2759 peaks called below 5% FDR and above 5-fold enrichment.<br>Brg1-Male-Nons: 36170 peaks called below 5% FDR and above 5-fold enrichment.<br>Brg1-Male-Smarcc1-6: 16681 peaks called below 5% FDR and above 5-fold enrichment.
Software	Seqmonk was used for all analysis.

Flow Cytometry

Plots

Confirm that:

☒ The axis labels state the marker and fluorochrome used (e.g. CD4-FITC).

☒ The axis scales are clearly visible. Include numbers along axes only for bottom left plot of group (a 'group' is an analysis of identical markers).

☒ All plots are contour plots with outliers or pseudocolor plots.

☒ A numerical value for number of cells or percentage (with statistics) is provided.

Methodology

Sample preparation	Xmas ESCs were prepared in KDS-BSS with 2% (v/v) FBS, with dead cells and doublets excluded by size.<br><br>Hematopoietic stem and progenitor cells (LSK: Lineage- Sca1+ c-Kit+ cells) were isolated from fetal livers from E14.5 Xmas female embryos, essentially as described107. Dissociated fetal liver cells were incubated with rat monoclonal anti-Ter119 antibody, then mixed with BioMag goat-rat IgG beads (Qiagen) and Ter119+ cells were depleted using a Dynal magnet (Invitrogen). The remaining cells were stained with Alexa700-conjugated antibodies against lineage markers Ter119, B220, CD19, Gr1, CD2, CD3 and CD8, APC-conjugated anti-c-kit/CD117 (generated by the WEHI Antibody Facility) and PE-Cy7-conjugated anti-Sca1 (BD Pharmingen). Cells were stained with FluoroGold to assess viability
Instrument	BD LSRFortesssa or BD FACSAriaIII
Software	Flow cytometry data were analysed using FlowJo.
Cell population abundance	Xmas mESCs are a pure population and therefore, once dead cells are excluded, they represent 100% of the population.
Gating strategy	Dead cells and doublets excluded by FSC and SSC.

☒ Tick this box to confirm that a figure exemplifying the gating strategy is provided in the Supplementary Information.
